# Surface Activation of Zirconia Orthodontic Brackets by Multi-Gas Atmospheric Plasma: Effects on Shear Bond Strength

**DOI:** 10.3390/ma19173564

**Published:** 2026-08-22

**Authors:** Ryota Okubo, Peng Chen, Taiki Osawa, Akitoshi Okino, Hiroyasu Kanetaka

**Affiliations:** 1 Division of Orthodontics and Dentofacial Orthopedics, Graduate School of Dentistry, Tohoku University, Sendai 980-8575, Miyagi, Japan; 2 Division of Interdisciplinary Co-Creation (ICC-Division), Liaison Center for Innovative Dentistry, Graduate School of Dentistry, Tohoku University, Sendai 980-8575, Miyagi, Japan; peng.chen.b7@tohoku.ac.jp; 3 Laboratory for Future Interdisciplinary Research of Science and Technology (FIRST), Institute of Integrated Research (IIR), Institute of Science Tokyo, Yokohama 226-8501, Kanagawa, Japan; 4 Institute of Biomedical Engineering (BME), Institute of Science Tokyo, Yokohama 226-8501, Kanagawa, Japan; 5 Division of Advanced Dental Science and Technology, Graduate School of Biomedical Engineering, Tohoku University, Sendai 980-8579, Miyagi, Japan

**Keywords:** zirconia, multi-gas plasma, atmospheric-pressure plasma jet, surface treatment, shear bond strength, orthodontic brackets, dental biomaterials

## Abstract

Zirconia orthodontic brackets exhibit favorable mechanical and esthetic properties; however, their chemically inert surfaces can limit adhesion to resin cements. This study evaluated the effect of multi-gas atmospheric plasma irradiation on the shear bond strength (SBS) of zirconia brackets. Zirconia brackets were treated with nitrogen (N_2_), argon (Ar), or air plasma for 3 and 10 s and compared with untreated controls and conventional alumina-sandblasted specimens. Surface wettability was assessed by contact angle measurements, and surface chemical changes were analyzed using Fourier transform infrared (FT-IR) spectroscopy. After 10 s of plasma treatment, the water-contact angle decreased from 64.3° ± 8.4° in the untreated group to 17.9° ± 6.4°, 18.8° ± 2.7°, and 16.8° ± 5.3° with Ar, N_2_, and air, respectively. For SBS testing, zirconia brackets were bonded to bovine enamel, stored in distilled water at 37 °C for 24 h, and subsequently tested (n = 12–15 per group). The mean SBS was 20.99 ± 2.87 MPa for the untreated group, 25.50 ± 2.40 MPa for the sandblasted group, and 23.63 ± 3.33, 23.99 ± 3.26, and 23.98 ± 3.58 MPa after 10-s Ar, N_2_, and air plasma treatment, respectively. FT-IR revealed no new absorption peaks. Within the limitations of this study, multi-gas atmospheric plasma treatment markedly reduced the apparent water-contact angle measured on flat zirconia specimens, whereas its effect on SBS relative to the untreated group was not statistically significant.

## 1. Introduction

Orthodontic treatment depends on reliable bonding between brackets and enamel to ensure effective force transmission throughout the course of treatment [1,2]. Inadequate bond strength may lead to bracket debonding, prolonged treatment duration, and an increased need for chairside interventions. However, excessively strong bonding can complicate bracket removal and increase the risk of enamel damage. Therefore, achieving a stable and clinically acceptable bond strength remains a key objective in orthodontic materials research. Traditionally, stainless steel brackets have been widely used because of their mechanical robustness and reliable bonding performance [3,4]. However, increasing patient demand for improved esthetics has driven the development of ceramic orthodontic brackets. In parallel, metal-oxide-based surfaces have attracted considerable interest as dental biomaterials because of their favorable physicochemical and biological properties [5]. Among these materials, zirconia has attracted considerable attention because of its superior mechanical properties, high fracture toughness, chemical stability, and favorable biocompatibility [2]. In addition, its tooth-colored appearance makes it particularly suitable for esthetic orthodontic applications. Despite these advantages, the intrinsically low surface reactivity of zirconia presents significant challenges for adhesive bonding [2,6,7,8]. Unlike metallic substrates, which can achieve mechanical retention through surface roughening, zirconia is chemically inert and exhibits low surface energy. These characteristics hinder resin wetting and limit interfacial adhesion. Consequently, various surface treatment strategies have been proposed to enhance the bonding performance of zirconia [7].

Conventional approaches include airborne-particle abrasion (sandblasting), tribochemical silica coating, and laser surface texturing [1,9,10,11,12]. Among these, sandblasting remains one of the most widely used clinical surface treatment methods. It enhances bond strength primarily by increasing the surface roughness and promoting micromechanical interlocking. However, abrasive treatments may introduce surface defects, microcracks, or stress concentration sites, potentially compromising long-term mechanical reliability [13,14]. Moreover, mechanical modification is irreversible and may alter the structural integrity of bracket surfaces. From a surface engineering perspective, an alternative strategy is to modify the surface energy and chemical reactivity rather than the surface morphology. Plasma-based surface activation has emerged as a promising noncontact technique capable of modifying the outermost surface layer of a material without affecting its bulk properties [15,16]. Plasma treatment can remove organic contaminants [17] from the surface, introduce polar functional groups, and enhance surface wettability. Importantly, these modifications are typically confined to the outermost nanometer-scale surface layer, thereby preserving the underlying crystal structure.

Atmospheric-pressure plasma systems have attracted increasing attention because of their operational simplicity and compatibility with clinical environments [18,19,20,21]. Unlike vacuum-based plasma systems, atmospheric-pressure plasma devices allow direct surface treatment under ambient conditions [16]. Furthermore, multi-gas plasma systems enable the modulation of the composition of reactive species by varying the feed gas such as argon, nitrogen, or air. Each feed gas generates a distinct profile of reactive species; however, the extent to which the gas composition influences the surface activation of zirconia remains unclear.

Previous studies have demonstrated that plasma treatment can enhance the bonding performance of zirconia used in restorative dentistry [17,22]. However, studies focusing specifically on zirconia orthodontic brackets remain limited, despite their unique surface geometries and bonding requirements. Furthermore, systematic comparisons of different plasma feed gases and irradiation durations are still lacking. In addition, most existing studies emphasize improvements in bond strength without thoroughly examining the relationship between surface chemistry, wettability, and mechanical performance. A comprehensive understanding of the correlation between plasma-induced surface activation and bonding behavior is essential for optimizing the treatment parameters.

Therefore, this study systematically investigated the surface activation of zirconia orthodontic brackets using a multi-gas atmospheric-pressure plasma system. Surface roughness, Fourier transform infrared (FT-IR) spectra, water-contact angle, and shear bond strength were evaluated to examine plasma-induced surface modification and the bonding performance of resin cements. By comparing untreated, sandblasted, and plasma-treated specimens under different gas conditions and irradiation durations, this study aimed to evaluate the effect of short-duration plasma treatment on interfacial adhesion.

## 2. Materials and Methods

### 2.1. Zirconia Brackets and Surface Treatment

Commercially available polycrystalline zirconia orthodontic brackets (COBY Pearl Color Ceramic Bracket; Biodent Co., Ltd., Tokyo, Japan), currently used in clinical orthodontic practice, were used in this study. The brackets feature a twin design with a mechanically retentive base and are available in 0.018- and 0.022-inch slot sizes. In this study, a 0.022-inch slot size was used. According to the manufacturer’s specifications, the bracket base dimensions were 3.4 × 3.8 mm, corresponding to a projected bonding area of 12.92 mm^2^, which was used for the shear bond strength (SBS) calculations.

Surface modification was performed using an in-house-developed multi-gas atmospheric-pressure plasma jet system, as described previously [17]. Plasma was generated between two electrodes under atmospheric conditions using argon (Ar; purity ≥ 99.999%), nitrogen (N_2_; purity ≥ 99.995%), or compressed air as the working gas. The gas flow rate was maintained at 3.0 L/min. A high voltage of 9 kV with a frequency of 16 kHz was applied to generate plasma with an output power of approximately 10 W. The plasma nozzle was positioned 3 mm above the zirconia bracket surface, and irradiation was performed for 3 or 10 s under each gas condition. All treatments were performed at 25 °C without vacuum assistance.

For comparison, alumina sandblasting, a widely used surface treatment for improving zirconia bonding, was used as a positive control. The bracket bases were sandblasted with 50 μm alumina particles using a sandblaster (Hiblaster Ovarjet, Shofu, Kyoto, Japan) at 0.3 MPa for 10 s at a distance of 10 mm. After sandblasting, the specimens were ultrasonically cleaned (US-105N; SND Co., Ltd., Nagano, Japan) and air-dried.

### 2.2. Surface Characterization

The surface characterization protocols applied in this study, including three-dimensional surface roughness analysis, contact angle measurement, and FT-IR spectroscopy, were based on the procedures used in our previous investigations [23]. Surface roughness and FT-IR measurements were performed on the zirconia bracket bases. Because of the limited area of the bracket base, flat zirconia specimens supplied by the same manufacturer and made from the same zirconia material as the brackets were used for water-contact-angle measurements. Surface characterization and bonding procedures were performed immediately after plasma treatment.

#### 2.2.1. Surface Roughness

Surface roughness was evaluated using a noncontact three-dimensional (3D) profiler (K505-232-02; Taylor Hobson Ltd., Leicester, UK) equipped with image analysis software (TalyMap Gold 6.2.6746). Three independently treated specimens were analyzed per group, and five randomly selected areas (300 μm × 300 μm) were measured for each specimen. The five measurements obtained for each specimen were averaged to obtain one representative value per specimen (n = 3).

#### 2.2.2. Surface Wettability

Static contact angle measurements were performed using a sessile drop instrument (PG-X-Plus; Matsubo Corp., Tokyo, Japan) equipped with a digital camera and image analysis software. Immediately after surface modification, 5 μL droplets of ultrapure water were placed on the treated surfaces. Three independently treated specimens were examined in each group. Five measurements were obtained from each specimen, and the mean value was calculated to obtain one representative value per specimen (n = 3). A preliminary time-dependent contact-angle assessment was performed using N_2_ plasma, with three independently treated specimens examined at each irradiation time point according to the measurement protocol described above. Based on this assessment, treatment durations of 3 and 10 s were selected for the subsequent comparison of Ar, N_2_, and air plasma treatments.

#### 2.2.3. Reflection-Mode FT-IR Spectroscopy

Reflection-mode FT-IR spectra were acquired using an FT/IR-4X spectrometer equipped with an RF-81S reflectometer (JASCO Corporation, Tokyo, Japan). Spectra were recorded over the wavenumber range of 4000–400 cm^−1^ at a spectral resolution of 4 cm^−1^ and subsequently subjected to baseline correction. Three independently treated specimens were examined for each condition, with one FT-IR spectrum acquired from each specimen. As the three spectra showed essentially indistinguishable spectral profiles, one of the three spectra was selected at random and presented as a representative trace.

#### 2.2.4. SBS Test

The SBS between zirconia orthodontic brackets and enamel was evaluated using bovine mandibular incisors as bonding substrates. Fifteen specimens were prepared for each group. To minimize variation associated with preparation time and manual handling, all specimens were prepared and bonded on the same day in five consecutive working sets, with three specimens per group processed in each set. These working sets were logistical subdivisions of the preparation procedure and did not represent independent experimental runs. Freshly extracted bovine mandibular incisors were purchased from Tokyo Shibaura Zouki Co. (Tokyo, Japan). All teeth were visually inspected to confirm the absence of cracks, defects, or surface irregularities. Eligible teeth were taken from the common specimen pool without preselection based on their visible characteristics and allocated to the experimental groups. No formal randomization sequence was used.

The roots were sectioned at the cemento–enamel junction using a precision band saw (K-100 Band Saw; HOZAN Co., Osaka, Japan). The pulp tissue was carefully removed using a dental explorer to eliminate any internal organic remnants. The labial enamel surfaces were flattened and standardized using a polishing machine (Marumoto Struers S5629; Marumoto Struers K.K., Tokyo, Japan) with waterproof silicon carbide abrasive paper (#600 grit; Marumoto Struers K.K.) under continuous water irrigation to prevent overheating. After surface preparation, each tooth was positioned with the labial enamel surface facing downward in a cylindrical resin mold (Sample Cup; Aoba Science Co., Tokyo, Japan) and embedded in a self-curing acrylic resin (Unifast II; GC Corporation, Tokyo, Japan). After complete polymerization, the exposed enamel surfaces were re-polished using the same polishing system to ensure uniform bonding surfaces and reproducibility among the specimens.

The enamel surfaces were etched with dental etching gel, thoroughly rinsed with water, and air-dried. Ortho Solo™ orthodontic bonding primer (Ormco Corporation, Brea, CA, USA) was then applied to the etched enamel surfaces using a disposable brush. Enlight™ light-cured orthodontic adhesive (Ormco Corporation) was applied to the bracket base. Each bracket was subjected to a static load of 10 N using a constant-load device. Excess adhesive was removed before light curing. Light curing was performed for 10 s using an LED curing unit (G-Light Prima 2 Plus; GC Corporation, Tokyo, Japan). All bonding procedures were performed by a single operator to minimize procedural variability. After bonding, the specimens were stored in distilled water at 37 °C for 24 h prior to mechanical testing. SBS [17] was measured using a universal testing machine (Autograph AG-I 20 kN, Shimadzu, Kyoto, Japan). A shear load was applied parallel to the bracket–enamel interface at a crosshead speed of 1.0 mm/min until failure. The maximum debonding load (N) was recorded and divided by the bracket base area (12.92 mm^2^) to calculate the shear bond strength (MPa). Fifteen tooth–bracket assemblies were initially prepared for each group. Specimens that detached before mechanical loading were recorded as unavailable measurements because no failure load could be obtained. Because the cause of detachment could not be reliably distinguished between insufficient bonding and incidental factors during storage or handling, these specimens were excluded from the primary quantitative analysis, and their numbers were reported separately. SBS values were obtained from 114 of the 120 assemblies, with final sample sizes of n = 15, 15, 14, 15, 12, 15, 13, and 15 for the control, sandblasted, Ar-3 s, Ar-10 s, N_2_-3 s, N_2_-10 s, Air-3 s, and Air-10 s groups, respectively.

#### 2.2.5. Statistical Analysis

No formal a priori sample-size calculation was performed; the SBS sample size of 15 specimens per group was determined pragmatically based on specimen availability and the planned same-day preparation workflow. Independently treated brackets or bonded tooth–bracket assemblies were considered the experimental units. Multiple measurements obtained from the same specimens were averaged before statistical analysis. Surface characterization data and descriptive SBS values are reported as the mean ± standard deviation. SBS data are presented as individual values together with the mean and 95% confidence interval. For the contact-angle data, differences among the three gas groups were analyzed separately at each treatment duration using one-way analysis of variance. For SBS, homogeneity of variance was assessed using Levene’s test. Because the group sizes and variances were unequal, differences among the eight groups were evaluated using Welch’s one-way analysis of variance followed by Games–Howell pairwise comparisons. The six plasma-treated groups were additionally examined using a two-factor linear model including gas type, irradiation duration, and their interaction, with HC3 heteroscedasticity-consistent covariance estimates. A sensitivity analysis was also performed by assigning an SBS value of 0 MPa to the six specimens that detached before mechanical testing. Contact-angle analyses were performed using JMP Pro, version 17 (SAS Institute Inc., Cary, NC, USA). SBS analyses were performed using custom scripts written in Python 3.12.13 with NumPy 2.3.5 and SciPy 1.17.0. HC3 heteroscedasticity-consistent covariance estimates were used to account for heteroscedasticity in the two-factor analysis.

## 3. Results

### 3.1. Surface Roughness Analysis

The three-dimensional surface roughness parameters of the zirconia brackets before and after plasma treatment are summarized in Table 1.

Compared with the untreated control group (Sa = 0.85 ± 0.43 μm), all plasma-treated groups exhibited higher arithmetical mean height (Sa) values. Ar-10 s and N_2_-10 s showed moderate increases (1.09 ± 0.13 μm and 1.16 ± 0.07 μm, respectively), whereas Air-10 s presented the highest Sa value (1.87 ± 0.49 μm). A similar trend was observed for Sq. Regarding the height distribution parameters, the Ssk values in the plasma-treated groups were closer to zero than those in the control group (0.60 ± 0.61). Sku values were approximately 2.0–2.2 in the treated groups compared with 3.50 ± 3.39 in the control group. The core roughness depth (Sk) increased after plasma treatment, with the highest value observed in the Ar-10 s group (4.07 ± 0.31 μm).

Overall, plasma treatment was associated with measurable changes in several surface roughness parameters. These observations are descriptive because only three independent specimens were examined per group and inferential comparisons were not performed.

### 3.2. Surface Wettability

**Figure 1:**
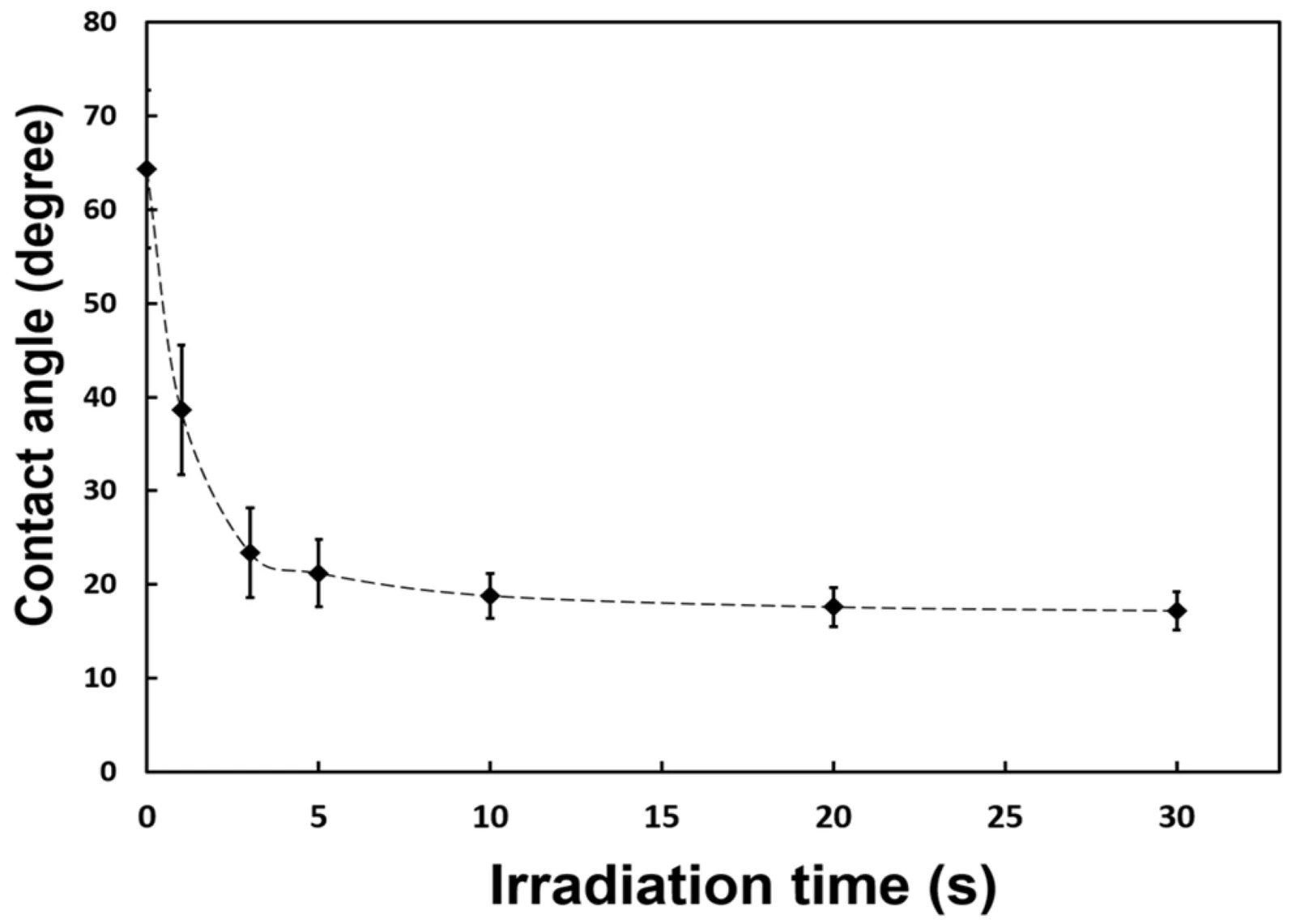
Time-dependent variation in water-contact angle after N_2_ plasma treatment. Data are presented as mean ± standard deviation (SD). The contact angle rapidly decreased within the first few seconds of plasma exposure and gradually approached a near-saturation state with longer treatment durations.

The effect of plasma irradiation time on surface wettability is shown in Figure 1. The untreated zirconia surface exhibited a relatively high water-contact angle (approximately 64°), indicating its moderate hydrophobicity. After plasma treatment, the contact angle decreased markedly within the first few seconds of exposure. Specifically, the angle decreased to approximately 38° after 1 s and further decreased to approximately 23° at 3 s.

**Figure 2:**
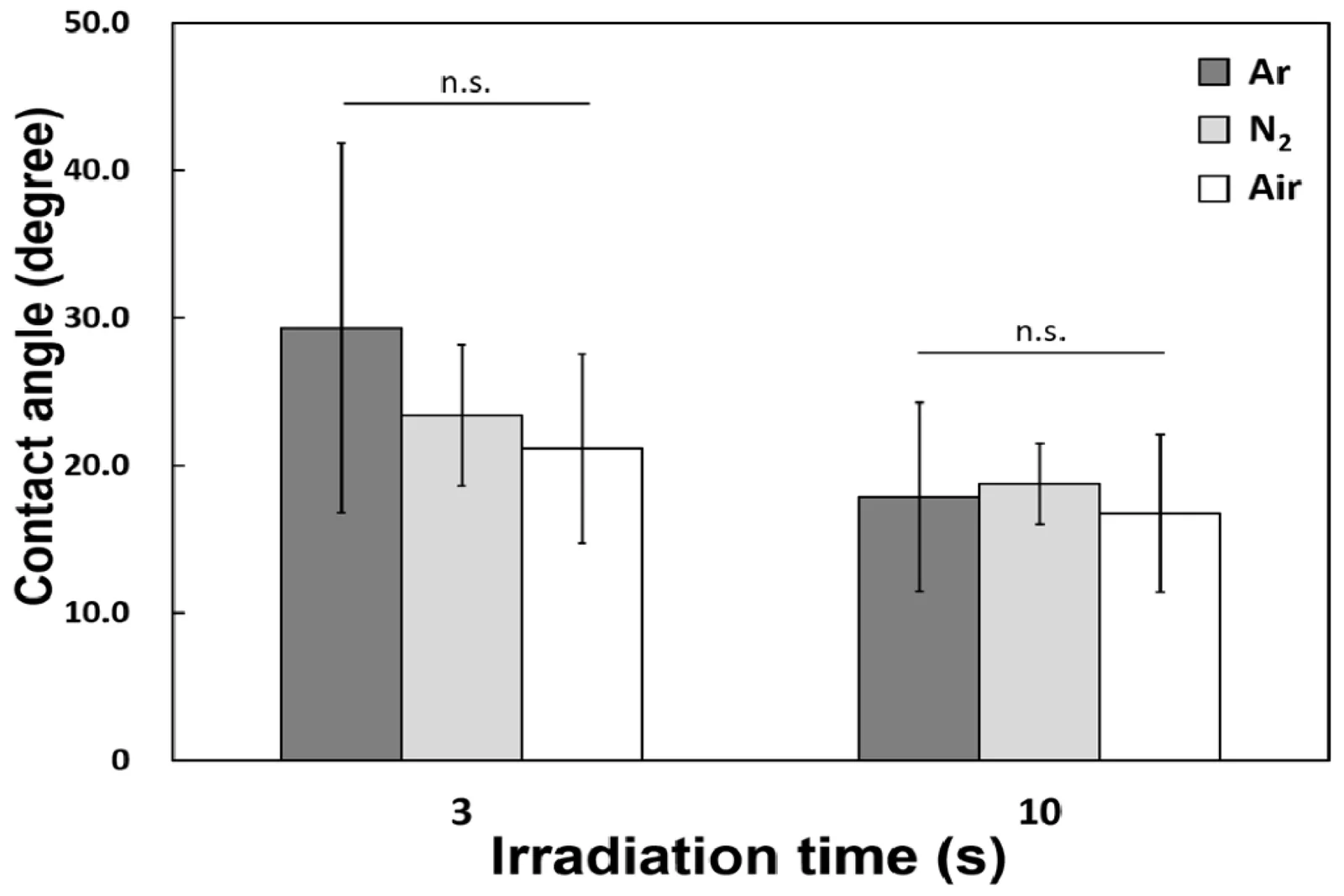
Comparison of water-contact angles after plasma treatment using different gases (Ar, N_2_, and air) at exposure times of 3 and 10 s. Data are presented as mean ± SD. No statistically significant differences were observed among the gas types for the same treatment duration. n.s., not significant.

With prolonged treatment (5–10 s), the contact angle showed only a minor additional reduction and stabilized within the range of 17°–20°, indicating near-saturation behavior. As shown in Figure 2, no statistically significant differences were observed among the Ar, N_2_, and air groups at either 3 or 10 s. Although small numerical variations were observed, the small sample size limits the ability to draw definitive conclusions regarding comparability among the different gas types.

These results demonstrated that short-duration plasma irradiation was sufficient to achieve a substantial reduction in the water-contact angle.

### 3.3. FT-IR Analysis

**Figure 3:**
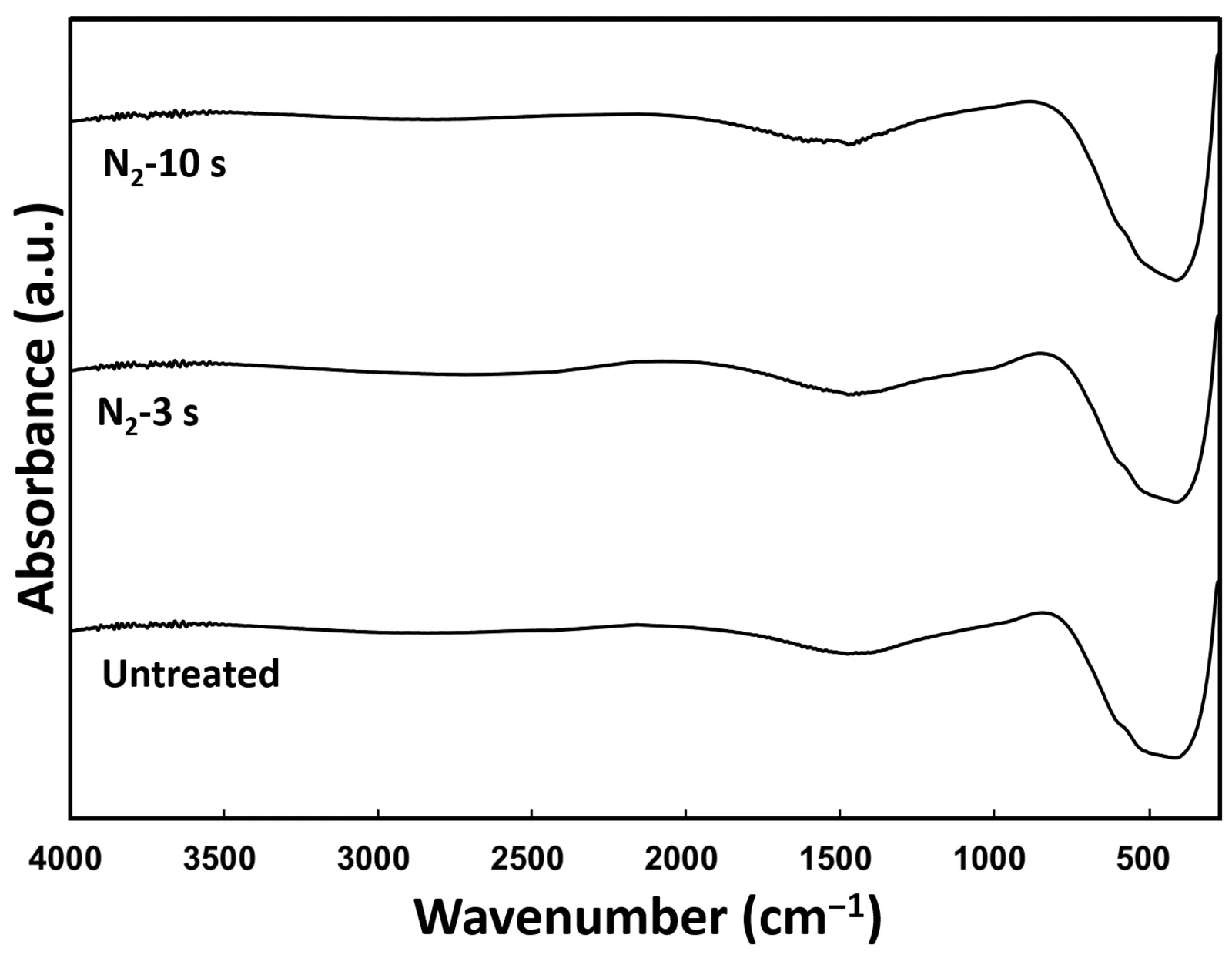
Representative reflection-mode Fourier transform infrared (FT-IR) spectra of untreated and N_2_ plasma-treated samples (3 and 10 s). The spectra are presented as absorbance and were baseline-corrected and vertically offset for clarity. a.u., arbitrary units.

The FT-IR spectra of the untreated and N_2_ plasma-treated zirconia surfaces (3 and 10 s) are presented in Figure 3. The overall spectral profiles of all groups were comparable across the measured wavenumber range.

In the low-wavenumber region (~400–700 cm^−1^), all specimens exhibited characteristic absorption bands associated with Zr–O lattice vibrations. These bands were consistently observed in both untreated and plasma-treated samples, with no noticeable peak shifts or changes in band shape detected after plasma irradiation. In the mid-wavenumber region (~1000–1600 cm^−1^), a slight decrease in band intensity was observed in both the 3 s and 10 s treatment groups compared with the untreated control. Although minor differences in spectral intensity were identified, the overall band positions remained similar among the groups. Importantly, no new absorption peaks emerged after plasma treatment at either exposure time. Collectively, these results indicate that short-duration N_2_ plasma irradiation resulted in subtle spectral intensity variations without inducing detectable peak shifts or the emergence of additional absorption bands. These results describe changes detectable by reflection-mode FT-IR and do not exclude chemical alterations confined to the outermost nanometer-scale surface layer.

FT-IR measurements were performed after treatment with each working gas. Because no newly detectable absorption peaks were observed under any condition, the N_2_-treated specimens are presented as representative results.

### 3.4. SBS

**Figure 4:**
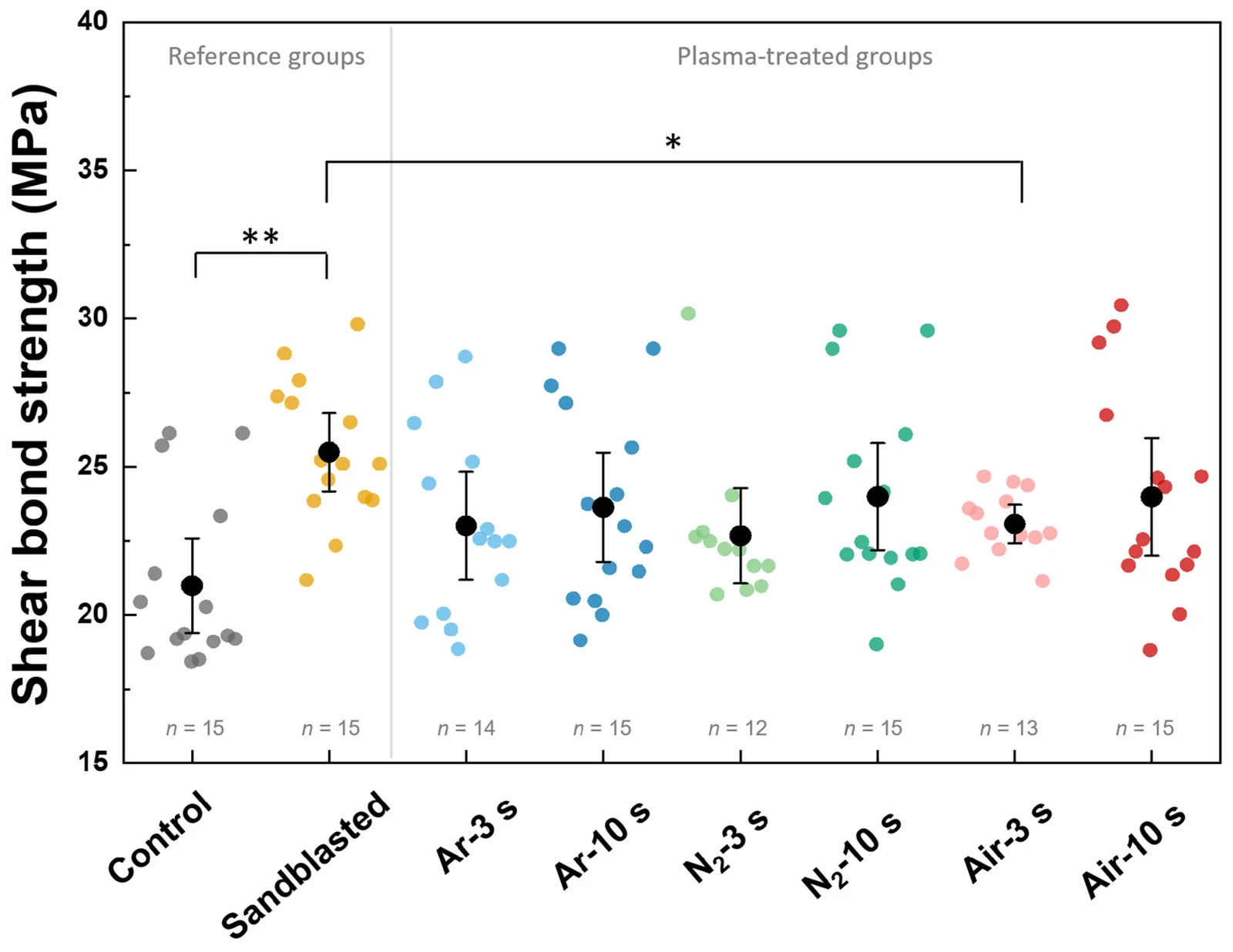
Shear bond strength (SBS) values of specimens after different surface treatments. Colored circles represent individual measured values, whereas black circles and error bars indicate the mean and 95% confidence interval, respectively. Differences among the groups were evaluated with Welch’s one-way analysis of variance, followed by Games–Howell pairwise comparisons. ** *p* = 0.0017 for control versus sandblasted; * *p* = 0.0367 for sandblasted versus Air-3 s. No other pairwise comparison reached statistical significance. Final sample sizes after specimens that detached before mechanical testing were excluded were n = 15, 15, 14, 15, 12, 15, 13, and 15 for the control, sandblasted, Ar-3 s, Ar-10 s, N_2_-3 s, N_2_-10 s, Air-3 s, and Air-10 s groups, respectively.

The SBS results for all the experimental groups are presented in Figure 4. The mean SBS values were 20.99 ± 2.87 MPa for the untreated control group and 25.50 ± 2.40 MPa for the sandblasted group.

All plasma-treated groups exhibited numerically higher mean SBS values than the untreated control group. The mean SBS values were 23.01 ± 3.14 MPa for Ar-3 s, 23.63 ± 3.33 MPa for Ar-10 s, 22.67 ± 2.54 MPa for N_2_-3 s, 23.99 ± 3.26 MPa for N_2_-10 s, 23.07 ± 1.08 MPa for Air-3 s, and 23.98 ± 3.58 MPa for Air-10 s.

Six pre-test detachments occurred exclusively in the 3-s plasma groups: 1/15 (6.7%) in the Ar-3 s group, 3/15 (20.0%) in the N_2_-3 s group, and 2/15 (13.3%) in the Air-3 s group. No pre-test detachments occurred in the 10-s plasma groups or the control groups. Levene’s test indicated heterogeneity of variance among the groups (*p* = 0.025). Welch’s one-way analysis of variance demonstrated a significant overall group difference (F(7, 44.41) = 3.289, *p* = 0.00665). Games–Howell comparisons identified significant differences between the untreated control and sandblasted groups (mean difference, 4.51 MPa; simultaneous 95% CI, 1.34–7.68 MPa; *p* = 0.00165) and between the sandblasted and Air-3 s groups (mean difference, 2.42 MPa; simultaneous 95% CI, 0.10–4.74 MPa; *p* = 0.03666). No other pairwise comparison reached statistical significance, and no plasma-treated group differed significantly from the untreated control.

In the heteroscedasticity-robust two-factor analysis of the six plasma-treated groups, no significant main effect of gas type (F(2, 78) = 0.048, *p* = 0.953), irradiation duration (F(1, 78) = 2.084, *p* = 0.153), or gas-by-duration interaction (F(2, 78) = 0.085, *p* = 0.919) was detected.

In the sensitivity analysis assigning an SBS value of 0 MPa to the six specimens that detached before testing, the overall group difference remained significant (Welch’s F(7, 47.51) = 4.121, *p* = 0.00131); however, only the difference between the untreated control and sandblasted groups remained significant.

## 4. Discussion

The present study systematically evaluated the surface activation of zirconia orthodontic brackets using a multi-gas atmospheric plasma system and examined the changes in surface roughness, surface chemical characteristics, wettability, and SBS. Short-duration plasma irradiation markedly improved surface wettability and produced numerically higher mean SBS values than the untreated control, although none of the plasma-treated groups differed significantly from the control. The concurrent changes in wettability, surface topography, and mean SBS are compatible with a possible contribution from improved interfacial wetting; however, the present results do not establish a causal mechanism.

### 4.1. Topographical and Spectroscopic Changes After Plasma Treatment

One of the key considerations when modifying zirconia surfaces for clinical applications is the preservation of bulk mechanical integrity. Zirconia is widely used in orthodontics because of its high strength, fracture toughness, and esthetic properties. However, conventional surface treatments, such as sandblasting, improve bonding primarily through mechanical roughening, which may introduce surface defects or microcracks that potentially compromise long-term reliability [13,14]. In the present study, the plasma treatment produced measurable changes in several surface-roughness parameters, particularly after 10 s of air-plasma treatment.

Although the mean Sa and Sk values were numerically higher in the plasma-treated groups than in the untreated control, the magnitude of these changes varied among plasma treatment conditions. A decrease in the mean Ssk toward zero indicates reduced positive skewness of the height distribution. The decrease in mean Sku toward approximately 2 indicates a flatter, platykurtic distribution and should not be interpreted as evidence that the surface became smoother because Sa and Sq increased simultaneously. Given the small sample size and the large variability in Ssk and Sku in the control group, these findings should be interpreted descriptively. Sandblasting was used as a conventional positive control for the SBS test. However, because surface roughness measurements were not obtained for the sandblasted brackets, the present topographical analysis was limited to comparisons between the untreated and plasma-treated groups, and no direct morphological comparison between plasma treatment and sandblasting could be made.

Reflection FT-IR analysis revealed no new detectable absorption bands or marked shifts in the principal Zr–O-associated bands after N_2_ plasma treatment. These observations suggest that no major FT-IR-detectable chemical changes occurred under these treatment conditions. However, the absence of new peaks does not demonstrate that the outermost surface chemistry remained unchanged because reflection-mode FT-IR is not specific to the nanometer-scale surface layer modified by plasma. Previous X-ray diffraction (XRD) and surface chemical analyses have suggested that short-duration atmospheric-pressure plasma irradiation predominantly modifies the outermost zirconia surface without substantially altering its crystalline phase [17]. Taken together, these findings indicate that short-duration plasma irradiation can modify the surface characteristics of zirconia brackets without abrasive treatment.

### 4.2. Potential Contribution of Enhanced Surface Wettability

The most pronounced effect observed in this study was the rapid reduction in the water-contact angle following plasma irradiation. The untreated zirconia surface exhibited moderate hydrophobicity, whereas plasma exposure markedly reduced the contact angle within a few seconds. Notably, most of the wettability improvement occurred within 3–5 s of irradiation, after which the contact angle reached a plateau. This time-dependent pattern indicates that most of the measurable changes in apparent wettability occurred within the first few seconds.

No statistically significant differences were detected among the gas groups for either treatment duration; however, the small sample size limits the interpretation of gas-dependent effects. As the reactive species generated by each gas condition were not directly characterized, the underlying gas-dependent mechanisms could not be determined. The increased apparent wettability may have facilitated adhesive spreading over the bracket base. However, adhesive spreading, interfacial voids, and load transfer were not directly assessed, and improved wettability should therefore be regarded as a potential contributing factor rather than a demonstrated mechanism. Notably, the contact angle approached a plateau after approximately 3–5 s, whereas the factorial SBS analysis identified no significant effect of irradiation duration, indicating that wettability alone cannot explain the observed numerical SBS pattern.

### 4.3. Relationship Between Surface Activation and Bond Strength

Sandblasting was included as a positive control because it is widely used to improve bonding to zirconia and provides reliable bond strength. As expected, the sandblasted group exhibited the highest mean SBS. None of the plasma-treated groups differed significantly from the untreated control, and no significant effect of gas type, irradiation duration, or their interaction was detected. Unlike sandblasting, which enhances bonding primarily through mechanical roughening and micromechanical interlocking, plasma treatment may improve interfacial wetting without abrasive surface modification. Plasma treatment may influence adhesive bonding through changes in apparent wettability, surface topography, and other unmeasured interfacial factors; however, the contribution of each factor could not be determined in this study.

Although several plasma-treated groups did not differ significantly from the sandblasted group, the absence of a significant difference does not establish equivalence or noninferiority. Plasma activation should therefore be considered a candidate surface-conditioning approach requiring further validation rather than a demonstrated alternative to sandblasting.

### 4.4. Clinical Implications

Orthodontic bracket use has shifted from metallic appliances to aesthetic materials such as ceramics and zirconia [3]. Although zirconia offers superior aesthetics and biocompatibility, achieving reliable bonding to enamel remains a clinical challenge. Traditionally, mechanical surface treatments have been employed to enhance bonding. However, these methods are irreversible and can affect surface integrity. Plasma activation can enhance surface wettability without relying on abrasive treatments [22]. The rapid improvement in apparent wettability suggests that atmospheric plasma may be compatible with short treatment times. However, bracket integrity, rebonding behavior, long-term durability, and clinical performance were not evaluated.

This study has several limitations. Bovine enamel was flattened and polished, and the SBS was evaluated only after 24 h of water storage without thermocycling, prolonged aging, or fatigue loading. Failure mode, adhesive remnant index, enamel damage, bracket fracture, adhesive spreading, and interfacial void formation were not evaluated. Six specimens detached before testing, and the sensitivity analysis indicated that one pairwise finding depended on how these specimens were handled statistically. Notably, all six pre-test detachments occurred in the 3-s plasma groups. Although this uneven distribution warrants caution when interpreting the SBS results, the causes of detachment could not be reliably determined. Surface characterization was performed using small numbers of specimens; the sandblasted surface was not characterized topographically; the outermost surface chemistry was not examined using a surface-sensitive technique; and no gas-flow-only control was included. Although the flat zirconia specimens were supplied by the same manufacturer and were made from the same zirconia material as the brackets, their surface condition may not have been fully identical to that of the actual bracket bases. In addition, the in-house plasma system was not fully characterized with respect to surface temperature, plasma-jet geometry, and reactive-species dose. Possible recovery of hydrophobicity after plasma treatment was not evaluated. These limitations preclude conclusions regarding the long-term clinical durability or equivalence to sandblasting.

## 5. Conclusions

Atmospheric-pressure low-temperature plasma jet treatment markedly reduced the apparent water-contact angle measured on flat zirconia specimens. The plasma-treated groups exhibited numerically higher mean SBS values than the untreated control; however, none of these differences reached statistical significance. The sandblasted group exhibited significantly higher SBS than the untreated control. In the complete-case factorial analysis of successfully tested specimens, no significant effect of gas type, irradiation duration, or their interaction was detected among the plasma-treated groups. However, because all pre-test detachments occurred in the 3-s groups, a possible association between shorter irradiation and pre-test detachment cannot be excluded. Further interfacial characterization and bond-durability testing are required before the clinical effectiveness of plasma treatment or its equivalence to sandblasting can be established.

## Figures and Tables

**Table 1:** Three-dimensional surface roughness parameters (ISO 25178 [24]) of ZrO_2_ with and without plasma treatment. Data are presented as the mean ± SD (n = 3).

Sample	Sa (μm)	Sq (μm)	Ssk	Sku	Sk (μm)
Control	0.85 ± 0.43	1.02 ± 0.49	0.60 ± 0.61	3.50 ± 3.39	2.76 ± 2.05
Ar-10 s	1.09 ± 0.13	1.28 ± 0.15	0.14 ± 0.07	2.10 ± 0.04	4.07 ± 0.31
N_2_-10 s	1.16 ± 0.07	1.36 ± 0.09	0.07 ± 0.07	2.00 ± 0.04	3.65 ± 0.55
Air-10 s	1.87 ± 0.49	2.24 ± 0.50	0.00 ± 0.02	2.19 ± 0.03	3.60 ± 0.62

Sa, arithmetical mean height; Sq, root-mean-square height; Ssk, skewness of the height distribution; Sku, kurtosis of the height distribution; Sk, core roughness depth derived from the material-ratio curve (ISO 25178).

## Data Availability

The data presented in this study are available on request from the corresponding author.
